# Patch Size, Functional Isolation, Visibility and Matrix Permeability Influences Neotropical Primate Occurrence within Highly Fragmented Landscapes

**DOI:** 10.1371/journal.pone.0114025

**Published:** 2015-02-06

**Authors:** Lucas Goulart da Silva, Milton Cezar Ribeiro, Érica Hasui, Carla Aparecida da Costa, Rogério Grassetto Teixeira da Cunha

**Affiliations:** 1 Institute of Natural Sciences, Federal University of Alfenas (UNIFAL-MG), Alfenas, Brazil; 2 Spatial Ecology and Conservation lab (LEEC), Department of Ecology, São Paulo State University (UNESP), Rio Claro, Brazil; The Australian National University, AUSTRALIA

## Abstract

Forest fragmentation and habitat loss are among the major current extinction causes. Remaining fragments are mostly small, isolated and showing poor quality. Being primarily arboreal, Neotropical primates are generally sensitive to fragmentation effects. Furthermore, primates are involved in complex ecological process. Thus, landscape changes that negatively interfere with primate population dynamic affect the structure, composition, and ultimately the viability of the whole community. We evaluated if fragment size, isolation and visibility and matrix permeability are important for explaining the occurrence of three Neotropical primate species. Employing playback, we verified the presence of *Callicebus nigrifrons*, *Callithrix aurita* and *Sapajus nigritus* at 45 forest fragments around the municipality of Alfenas, Brazil. We classified the landscape and evaluated the metrics through predictive models of occurrence. We selected the best models through Akaike Selection Criterion. Aiming at validating our results, we applied the plausible models to another region (20 fragments at the neighboring municipality of Poço Fundo, Brazil). Twelve models were plausible, and three were validated, two for *Sapajus nigritus* (Area and Area+Visibility) and one for *Callicebus nigrifrons* (Area+Matrix). Our results reinforce the contribution of fragment size to maintain biodiversity within highly degraded habitats. At the same time, they stress the importance of including novel, biologically relevant metrics in landscape studies, such as visibility and matrix permeability, which can provide invaluable help for similar studies in the future and on conservation practices in the long run.

## Introduction

Human activity, such as rapid urbanization and intense land use for agriculture and ranching, modify the original landscape, reducing the quantity, quality and spatial configuration of essential resources for the maintenance of the local and regional biodiversity [1–3]. As a consequence of such anthropic impact, habitat fragmentation intensifies [4], usually with negative effects for various taxa [5], [6]. Neotropical primates, being primarily arboreal, are profoundly affected by habitat loss and fragmentation, particularly within originally forest-dominated landscapes [7–9]. As these primates find food, shelter and reproductive sites in the trees, they are highly sensitive to changes in the vertical structure of the vegetation, as well as to the availability of such sites in the landscape [10], [11]. Given that primates are involved in complex ecological processes, such as dispersion, frugivory and predation [12–14], [9], landscape degradation negatively influences their population dynamics. As a consequence, the structure, composition and viability of the entire community can be directly or indirectly influenced by impact on primates [15], [7], [9]. Notwithstanding the importance of understanding which factors influence primate spatial distribution, few studies have focused on predicting primate occurrence [10]. Most of these studies relate primate presence mainly to fragment size and isolation (Euclidian distance), not taking into account ecological needs and abilities of the animals [16–18]. Understanding how different landscape aspects directly influence primates, which have direct and indirect effects on several ecological processes, is essential for defining more effective strategies for biodiversity conservation through landscape and resource management. We aimed at estimating the relative contribution of fragment size, functional isolation, visibility between neighbor fragments and matrix permeability on the occurrence of three Neotropical primate species in an Atlantic forest region.

Forest fragmentation is the process by which a former continuous habitat undergoes loss and subdivision into patches that are smaller, isolated, of a lower quality and under a stronger influence on the border with anthropic matrices (i.e. non-habitat, such as sugar cane, pasture, forestry etc. [19–22]). Most populations of forest animals living in these landscapes are more vulnerable not only to environmental, demographic and genetic stochastic events [23–25], but also to several other negative factors, such as endogamy [4], [26]. As a result, the distribution, diet, home range, population size and social organization are also negatively affected [27], [28]. Eventually, all those factors increase the likelihood of local extinctions [29], [30].

Primates are no exception, and several authors have observed negative effects of fragmented landscape parameters on arboreal primates (e.g. [31], [12], [28]). The negative effects of fragment size are the most commonly studied issue [32], [33]. Effects resulting from patch isolation are also commonly studied [31]. Such isolation, however, is usually measured via Euclidean distance, a metric which ignores several landscape elements, such as physical barriers, stepping stones, matrix types, buildings and, more importantly, the ability of species to transpose these landscape features. Moreover, the most common approach is to include in the analyses all fragments surrounding the focal ones, irrespective of their characteristics, thus not taking into account their real usefulness for the animals. In our study, we improved the isolation metric, by considering the size of the surrounding fragments as potential targets to be accessed, and also the presence of insurmountable barriers (e.g. reservoirs and cities). We therefore attempted to bring this variable to a more biologically meaningful measure for the studied species.

Matrix type is also another key landscape aspect that has been explored in population studies of arboreal primates [8]. However, to our knowledge, no study has focused on the permeability of the matrix regarding primate movement. We do not know, for example, if distinct matrix types influence primate movement differently. However, movement is a primary factor in the establishment and maintenance of scattered populations, and it must be considered when evaluating the contribution of habitat isolation or connectivity of forest fragments [28].

Landscape relief is another likely relevant, yet poorly explored, variable that may influence several animal species [34], [35], and has not been considered for primates so far. Primates most likely acquire knowledge about surrounding forest fragments through their vision, which is highly developed and largely employed in locomotion, foraging and social activities [36], [37]. Thus, how can a primate know about the existence of a neighboring fragment, if the relief obstructs an individual’s ability to see them? Therefore, taking into consideration the biological reality of primates facing rugged topography can help clarify part of their distribution in the landscape, by revealing the existence of “hidden” forest fragments.

Within the above context, the objective of this study was to answer the following questions: Is the size of forest fragments in a highly fragmented region an important parameter for predicting primate occurrence? Is our refined isolation metric plausible for explaining the occurrence of the studied species? Do structural differences among matrix types, focusing on animal permeability, influence primate occurrence in forest fragments? If so, is there a matrix type that is more favorable to primates? Are forest fragments that are “hidden” by natural physical barriers less accessed than visible ones?

## Material and Methods

### Study region and fragment selection

**Figure 1:**
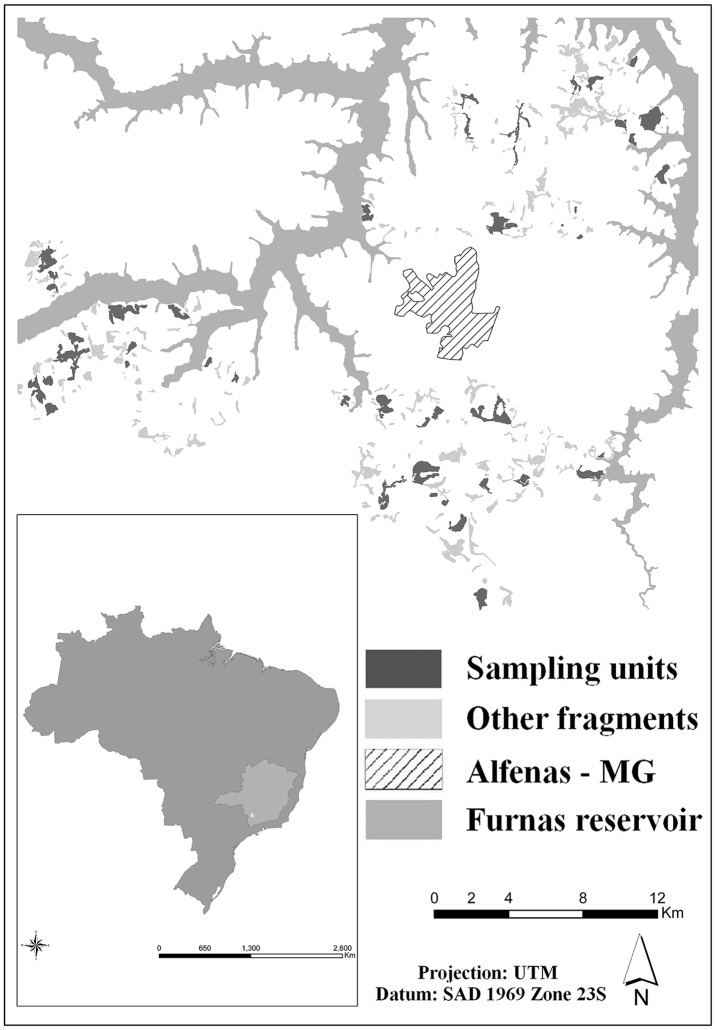
Focal study area, Alfenas—MG, Brazil. The Atlantic Forest remnants in the region are highly fragmented, with the vegetation reduced to 4% of the original area. The forest fragments were manually vectorized, scale 1:10,000, followed by visual grading. Figure originally produced by LGS based on Google Earth images, for illustrative purposes only.

We carried out the study in forest fragments around the municipality of Alfenas, Minas Gerais, Brazil (21° 27’ S / 45° 56’ W; Fig. 1), where the vegetation is highly degraded and fragmented, with roughly 4% remaining [38]. The remaining fragments have an average size of 10ha (0.1 to 182 ha). We selected 45 fragments for the study, encompassing a gradient of size and isolation. All fragments are comprised of secondary semideciduous forest. Regarding forest quality, an unpublished study, which evaluated fragment quality using linear spectral mixture model (soil, shadow and vegetation fractions) obtained that the forest´s quality of the region does not vary much (please note that no field surveys or studies were conducted to confirm this finding). If a previously chosen fragment proved to be highly degraded in the field, such as one in more initial succession phases, it was excluded from the set, and another was chosen. The entire area is a mosaic of small properties, and all fragments were located within private land. We obtained prior permission from landowners to access all areas where the study was conducted.

### Primate species

We studied three primate species which occurs in the region, *Callicebus nigrifrons* (black-fronted titi monkeys), *Callithrix aurita* (buffy-tufted-ear marmosets) and *Sapajus nigritus* (black capuchin monkeys):

- *Callicebus nigrifrons* is a medium-sized primate (1050 to 1650g; [39]), with a predominantly frugivorous diet [40]. It is endemic to the Atlantic forest, and are found on both primary and secondary forests, being well-adapted to the latter [39], [41];
- *Callithrix aurita* is a small-bodied primate (400 to 450g; [42]), also endemic to the Atlantic forest. It inhabits upland evergreen and semideciduous forest from 600 to 1200 m a.s.l. [43]. Differing from the more generalist species of the genus, such as *C. jacchus* and *C. penicillata*, *C. aurita* is more sensitive to fragmented habitats, given it is not well-adapted to exudate consumption, and insects are the main item of their diet [44]. The species is listed as vulnerable on the IUCN′s red list [45];
- *Sapajus nigritus* is a medium-sized primate (1.3 to 4.8kg; [46]) with vast geographic distribution, mostly within the Atlantic Forest biome (states of Minas Gerais, Espírito Santo, Rio de Janeiro, São Paulo, Paraná, Santa Catarina and Rio Grande do Sul), and reaching the northernmost tip of the Argentinian province of Misiones [47]. It is characterized by a way of foraging in the canopy labeled as destructive [48]. It is an omnivorous species, with a diet composed mainly of fruits, seeds, nectar and animal prey [49]. The species is also capable of consuming crops to complement its diet [50].

Given their occurrence in the Atlantic forest, a highly fragmented biome, *C. nigrifrons* and *S. nigritus* are listed as near threatened on IUCN′s red list [45].

### Ethics Statement

According to the bylaws of the Institutional Committee on the Ethics of Animal Use of the Universidade Federal de Alfenas, we did not need any formal authorization to conduct this study, as our project did not involve animal experimentation.

### Survey of primate occurrence

To record occurrence of primate species, we employed the playback sampling technique [51]. The sampling scheme involved broadcasting calls of each study species separately, both inside and at the border of each focal fragment. Inside the fragments, we reproduced calls in three different directions (120° from each other) at each sampling point, thus covering 360° around them; from fragment borders, we broadcasted calls in two directions, each one at a 45° angle in relation to the border line. We waited one minute in silence after each playback (adapted from [52]). If there was no response (vocal or approaching) after the calls at a given point, we repeated the procedure at another point. We sampled each fragment as many times as necessary to cover the majority of its area or until we obtained a positive response. Towards this end, we selected sampling points a priori on satellite images. These were separated by 200 m, and covered the whole fragment. Half of this value (100 m) was an estimated distance that the playback could reach with a minimum degree of degradation, although it can travel further. Recall that playbacks are broadcasted in all directions; thus, the minimum 100 m broadcast circles of two consecutive playbacks touch each other. Nonetheless, to ensure even better coverage, calls were played back at every 100 m, whenever possible. Some previously selected points were very difficult to reach, and were thus excluded. We estimate our sampling effort allowed us to cover at least 80% of the fragments. Distance between sampling points was always shorter than the estimated hearing range. If we did not obtain a positive response for one or more species in a given sampling session, we carried out two other visits to the fragment, at different dates, before considering the unresponsive species as being potentially absent from that focal fragment.

We carried out playbacks from July 2010 until January 2013. For each species, we selected the call type to be reproduced based on its behavior, choosing the one most likely to elicit responses. Such calls are those usually related to resource defense, group protection, or the maintenance of contact between group members. Thus, we chose long phee calls [53] for *C. aurita*, duets [54] for *C. nigrifrons* and contact whistles [55] for *S. nigritus*. The recordings played back to survey *C. aurita* were actually from *C. flaviceps*, a procedure successfully adopted in our research group. The calls for detecting *S. nigritus* came from *S. apella* [56], following the procedure recommended in [57]. Note that former *S. apella* has been split lately into several species, *S. nigritus* being one of them. The same calls were employed throughout the study.

To detect primate occurrence at the validation site (see below), we employed a similar methodology. There were minor differences though, aiming at speeding up data collection, while maintaining the coverage and the chance for a positive response. Playback locations were not selected a priori, but were chosen opportunistically. We first played back from the borders, and after circling the perimeter of the fragment, we crossed it. We estimate that the maximum distance between points was 200m. At the points within the fragments, calls were played back in four directions, not three. We waited five minutes between playbacks at a given location. Playbacks were carried out exclusively during mornings, when the chance of a response is increased, from our experience.

### Land cover, land use and landscape metrics

To select the focal fragments, we employed both Landsat satellite images from 2011 and 2012, and also a map of land use and cover available at a 1:50,000 scale. Then, for each landscape surrounding the focal fragments we produced a new land use and vegetation cover map. We generated these maps by manual vectorization, at a 1:10,000 scale, followed by visual classification, of high resolution (1 m) Google Earth images in the program QGIS 1.8, with the help of the OpenLayer plug-in. We also employed ArcGis 10.0 software to select the sampling fragments and calculate fragment metrics. For constructing the occurrence models and also for testing the plausible ones (see below) we calculated or estimated the following parameters for each fragment (Table 1—focal fragments; Table 2—validation fragments):

**Table 1:** Characteristics of the 45 focal fragments regarding the study metrics.

**Metric**		**MIN**	**AVG**	**MAX**
Area (ha)		0.92	30.29	96.21
Isolation (m)	*Callicebus nigrifrons*	2.90	367.74	1150.60
	*Callithrix aurita*	2.90	709.80	5500.00
	*Sapajus nigritus*	2.90	963.04	5500.00
Visibility (ha)		1.19	49.65	281.10
Matrix Permeability Index (MPI)	*C. nigrifrons*	0.73	9.42	39.82
	*C. aurita*	0.74	12.44	60.75
	*S. nigritus*	1.18	25.46	94.97

MIN—minimum value, AVG—average value, MAX—maximum value. Note that although the fragments are the same for all species, the average and maximum values for isolation and MPI are different among the species. This result occurred because the metrics include values that are ecologically calibrated that are different among the species.

**Table 2:** Characteristics of the 20 fragments employed for model validation, regarding the study metrics.

**Metric**		**MIN**	**AVG**	**MAX**
Area (ha)		4.03	43.14	164.24
Isolation (m)	*Callicebus nigrifrons*	12.87	326.43	1153.16
	*Callithrix aurita*	17.24	715.69	1153.16
	*Sapajus nigritus*	17.24	715.69	1153.16
Visibility (ha)		4.7	76.77	281.07
Matrix Permeability Index (MPI)	*C. nigrifrons*	4.28	18.59	39.89
	*C. aurita*	6.02	21.58	42.15
	*S. nigritus*	8.77	28.46	54.34

Codes and comments as in Table 1.

**Figure 2:**
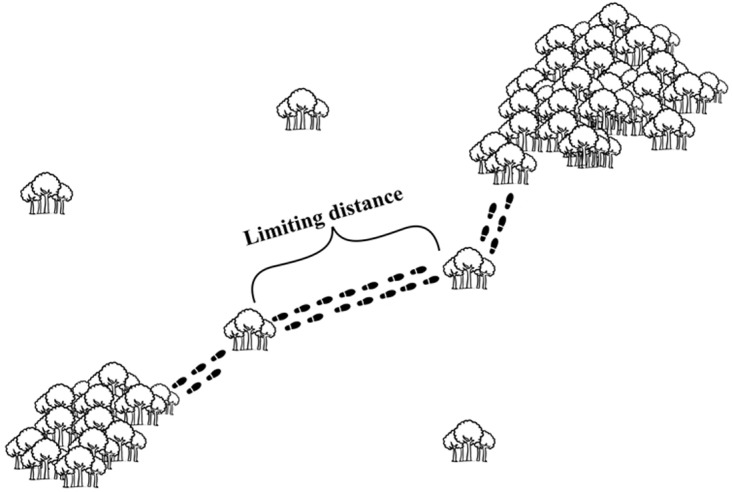
Illustration of the approach for calculating the isolation metric.

**Figure 3:**
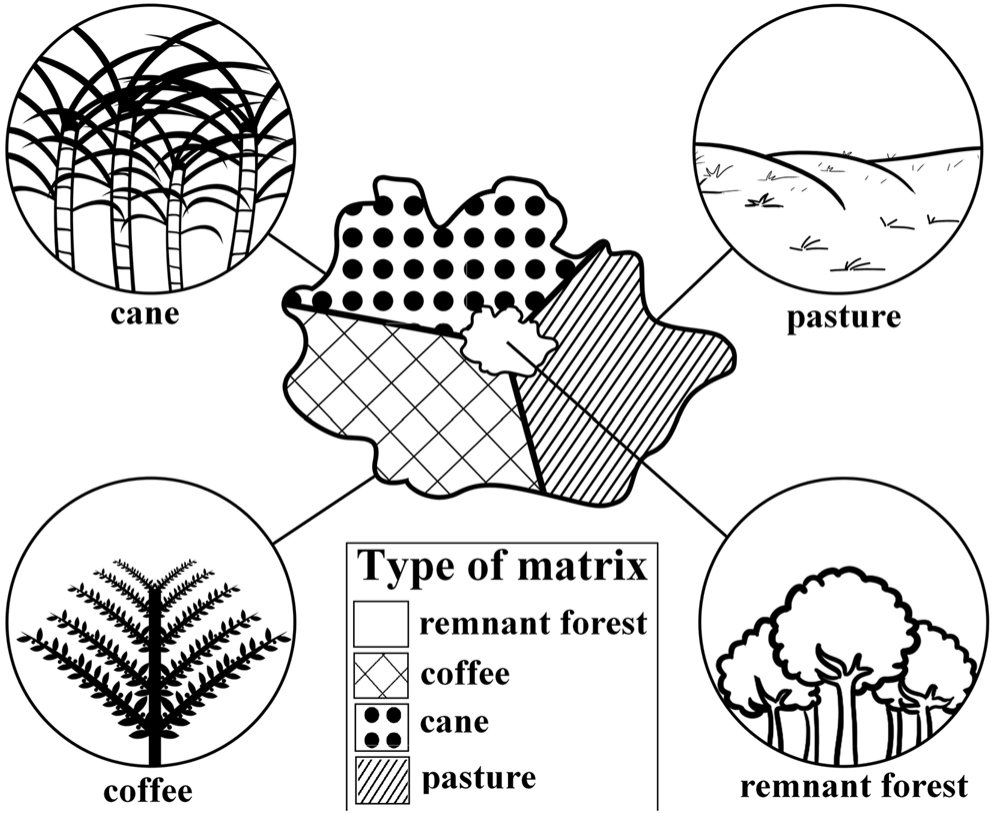
Representation of the structure of the three main types of matrices (sugarcane plantations, coffee plantations and pastures) found in the region of Alfenas-MG. The structure of the sample unit (forest remnant) was also represented.

- Area: size of the forest fragment, in ha;
- Functional isolation: we attempted to calculate an isolation metric which had a higher biological significance than the mere Euclidean distance between the focal fragments and their nearest neighboring fragment. Thus, we improved the isolation metric by simultaneously taking into account the presence of possible stepping stones, the size of the neighboring fragments (potential source or targets for dispersion) and the limiting traveling distance in the path between the sampling fragment and its neighbors. We considered as neighboring fragments all fragments surrounding the focal patches, including other sampled fragments, if any. Firstly, based on the sizes of the studied fragments and on the primate presence/absence data in them, we calculated an area that corresponds to a 50% chance of occurrence (probability = 0.50, hereafter p50) of each study species, based on the logistic regression curve of species presence against area. Thus, areas equal to or higher than the threshold p50 value for a given species have a 50% chance of bearing it. Then, from the entire set of neighboring patches of each focal fragment, we selected only those with a higher probability of harboring a certain primate species, that is, those with an area equal to or larger than p50. Note that this procedure was carried out independently for each species, since they differ in their p50 values. Thus, the sets of nearest neighbors differ for each species. Next, for each species we drew links between each of the 45 sampling fragments and their selected nearest fragments. Such paths, however, were not the shortest linear distances between the fragments (Euclidean distances). Instead, we chose multi-segmented, zigzagging routes that had the shortest distances over open areas. For example, in landscapes with stepping stones (such as fragments smaller than p50), the paths would include them if the route line segments were smaller than the straight distance between the focal fragment and the neighbor. In addition, we also included unavoidable detours, such as going around water bodies. Thus, the traveling rule was not: “walk in as straight a line as possible between the focal fragment and a neighbor”. Instead, the rule was: “walk along a route which includes the shortest line segments over open areas between the fragments and avoiding insurmountable barriers”. We then considered our functional isolation metric to be the largest distance (among the sequences of segments for each route) over an open area in a path between the focal fragment and its nearest p50 neighbor (Fig. 2). We believe this segment is the limiting factor in the movement between these neighboring fragments;

- Visibility, in ha: we proposed a new metric in order to quantify the summed area of visible forest remnants surrounding the focal fragments. We first superposed a land use and land cover map over a digital elevation model with the topography (IBGE; 1:50.000) of the region. Next, we traced lines of sight from the border of each sampling fragment to the border of each of its neighboring forest patches (commonly more than one line between each fragment pair) within a 500m buffer (drawn from the borders of the fragments). Towards that end we obtained the elevation values of all pixels under each line of sight using the *3D Analyst—Interpolate Line* tool from the ArcGis 10.0 software. We thus produced the topographic profile of such lines. If at least one of the profiles showed a clear line of sight from a focal fragment towards a neighboring one, the latter was deemed visible. We considered a profile to show a clear line of sight if, from a virtual tree, standing 10m high at the border of the focal fragment, a monkey could have a direct view of a similar tree standing at the border of the target fragment. The size of all visible fragments around a focal fragment was summed to calculate its ‘visibility’. We chose the 500m buffer criterion as a rough estimate of the maximum distance a primate would travel in an open area. Such information is extremely rare in the literature, and we based our criterion on the few available values [31], [58], [59]. The idea behind this new visibility metric is to add more realism to the standard metric of the percentage of forest cover surrounding a study area. We imagined that if two forest fragments are not visible from each other (something that is not readily apparent from 2D satellite images), then a monkey would not travel between them;
- Matrix permeability: there are three main matrix types in the study region, coffee, sugar cane and pastures. These types can be ranked from the most structurally similar to the original habitats (coffee plantations) to the more dissimilar one (pastures), and sugar cane being intermediary (Fig. 3). Broadly, we aimed at identifying whether coffee plantations are more permeable to movement than sugar cane plantations, and if both are more permeable than pastures, given their structural differences. However, information regarding the influence of permeability of each of these types on Neotropical primate movement are basically non-existent, and difficult to obtain first-hand. We then resorted to a methodology based on the knowledge of experts (adapted from [60]). We sent questionnaires to researchers with field experience on one or more of the study species, asking them to attribute a permeability value (PV) for each of several land use types for each species. Such values varied from 0 to 100, in increments of 10 units, 0 being the least and 100 the most permeable. Then, the PV for a given matrix type was the average of the values attributed to it by several researchers. Next, for each fragment we attributed a Matrix Permeability Index (MPI). The higher this index, the more permeable is the set of matrix types surrounding a focal fragment. The MPI was calculated according to this formula:
  $$\text{MPIb=}\frac{\sum \left(\text{PVs*As}\right)}{\text{Ab}}$$
  Where: MPIb = Matrix Permeability Index at point b; PVs = Permeability Value of land use type s; As = Area occupied by land use type s; Ab = Total area of the 500m buffer at point b; b = focal fragments; s = Land use type (coffee, sugar cane or pasture).

Note that this index, being a summation, takes into account the fact that each fragment is surrounded by more than one matrix type. Thus, every matrix type contributes to the MPI index in proportion to its percentage of coverage around the buffer.

### Competing models

To evaluate the influence of different environmental parameters on the occurrence of the study species, we generated a set of a priori competing models. Each model included a combination of landscape metrics that we considered relevant for the species (Table 3). We avoided comparing all possible combinations of landscape metrics, because we decided to only evaluate ecologically meaningful models [61].

**Table 3:** Ecological hypotheses associated with the multiple logistic regression models that were tested to explain the occurrence of *Callicebus nigrifrons*, *Callithrix aurita* and *Sapajus nigritus* in forest remnants in a highly fragmented landscape.

**Models**	**Hypotheses**
Species~Area	Small forest fragments offer limited resources, negatively affecting population survival and making them more vulnerable to local extinction.^1, 2^
Species~Isolation	Populations which are isolated from source patches (p50) have their long-term viability decreased due to genetic drift and increase in endogamy.^1, 5^
Species~Matrix Permeability	More permeable matrix types increase the possibility of resource acquisition and favor movement between neighboring fragments, thus increasing chance of population survival.^3, 4, 5^
Species~Visibility	Visible fragments are more easily accessed and contribute to the establishment of metapopulations. The higher the total area of visible fragments, the higher is the likelihood of population survival, through metapopulations.
Species~Area + Visibility	Both fragment size and the amount of available habitat around it are important for the maintenance of populations/metapopulations.
Species~Area + Matrix Permeability	Both parameters considered together are better than each one in isolation in explaining primate presence. Small fragments surrounded by a more permeable matrix habitat offer additional areas for resource acquisition. On the other hand, large fragments surrounded by a more inhospitable matrix are more isolated, with a higher chance of local extinctions.
Species~Area + Isolation	Small fragments that are closer to larger remnants can be viable.
Species~Matrix Permeability + Isolation	Matrix permeability partially compensates the effects of isolation. Thus, isolated fragments embedded in a permeable matrix are less functionally isolated.
Species~Matrix Permeability + Visibility	Fragments that are visible and connected by a permeable matrix contribute to the establishment of metapopulations.
Species~Null	Species occurrence is not affected by any of the parameters, being randomly determined.

^1.^ Fahrig 2003.

^2.^ Harcourt and Doherty 2005.

^3.^ Antongiovanni and Metzger 2005.

^4.^ Gascon et al 1999.

^5.^ Pozo-Montuy and Serio-Silva 2011.

### Data analysis

We employed univariate and multiple logistic regression models (binomial distribution, logit function) to evaluate the relation between presence/absence of each of the three species separately (*C. nigrifrons*, *C. aurita* and *S. nigritus*) and the four ecologically meaningful landscape metrics described above. We selected the plausible models based on the Akaike Criterion Information, corrected for small samples (AICc) [62], [63]. To avoid the usage of explanatory variables with high collinearity [64] we first performed a Pearson´s correlation analysis within R 2.15.3 software [65]. Only explanatory variables with Pearson´s correlation (r) < 0.7 were kept. As all our independent explanatory variables had r<0.7, they were all kept on the model building process.

We considered those models with the lowest AICc values (ΔAICc<2) as the more plausible ones. We also selected models with wAICc higher than 0.1 (i.e. > 10%) as plausible. The wAICc index allows for estimating the relative efficiency of a model, given a list of competing models attempting to explain the patterns in the data [61]. We also computed the contribution of the explanatory variables that appeared in the plausible models, by adding up the wAICc of all models in which these variables appear. In addition, we carried out an ANOVA (F test) and calculated the p-value for the best models of each species.

### Model validation

Following [66], we validated the plausible predictive models of occurrence (wAICc>0.1) using an independent dataset comprised by 20 landscapes centered at forest fragments in the municipality of Poço Fundo/Brazil (21°46′ S / 45°57′ W), located 50km from Alfenas (Table 2). First, we collected primate occurrence data on these fragments, employing the methodology as described above. We then calculated landscape metrics (patch size, functional isolation, visibility and matrix permeability) for each landscape, as we did for the 45 fragments that we used to build up the models. For each species, we estimated its probability of occurrence in each of the 20 validation landscapes, according to each of its plausible models. We adopted 80% as the threshold probability value for distinguishing presence from absence. Thus, landscapes with a probability of occurrence of a given species higher than 80% were predicted to harbor that species. We then compared such predictions with the field data, verifying which models successfully explained primate occurrence, and which did not. We generated the confusion matrix [67] and then calculated the kappa statistic [68] for such comparisons and for estimating the precision of each model. The confusion matrix is a 2 x 2 table, including the true and false positives and negatives. The kappa statistic [69] is a measure of model accuracy for the predictions. It takes into account the correspondence between observed and predicted values due merely to chance [67].

## Results

**Figure 4:**
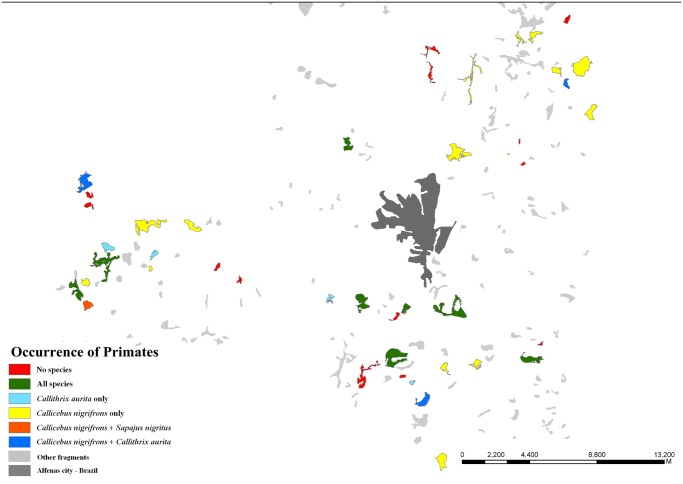
Presence/absence data for the three study species in the focal fragments. Figures originally produced by LGS based on Google Earth images, for illustrative purposes only.

Among the 45 surveyed fragments, we registered the occurrence of *C. nigrifrons* in 28, of *C. aurita* in 15, and of *S. nigritus* in nine of them (Fig. 4).

**Figure 5:**
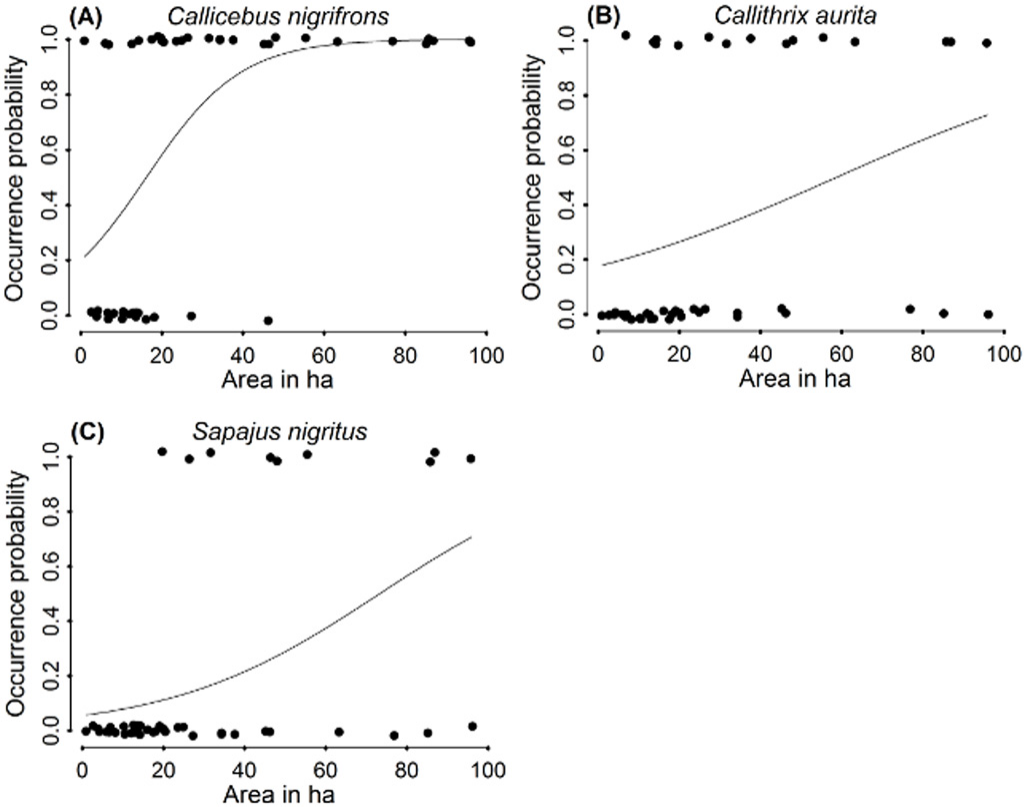
Fitted logistic regression curves for the occurrence of the studied primate species against best predictor, fragment area. (A) *Callicebus nigrifrons*, (B) *Callithrix aurita* and (C) *Sapajus nigritus*.

When evaluating competing predictive models (Table 3) for each species, fragment area appeared as the main variable explaining primate occurrence, more so for *C. nigrifrons* (Table 4). The same pattern can be observed if we consider the cumulative wAICc for the species (Table 5), with patch size showing a very high contribution to explain species presence/absence. Visibility, matrix permeability and isolation, when combined with patch size, were also important, as they appeared among the best models (ΔAICc<2)—Table 4. However, we consider these variables as secondary factors, as all of them showed cumulative wAICc around 0.20, independent of the species (Table 5). These explanatory landscape variables never appear isolated in plausible models, but always combined with the fragment area in less plausible models than those considering area alone (Table 4). Fitted logistic regression curves for the models containing fragment area only (Fig. 5), however, do not show curves as steep as expected, except for *C. nigrifrons*. Note, however, that small fragments most frequently do not harbor the species, while larger ones do so more frequently, particularly in the case of *C. nigrifrons*. The ANOVA results for the best models for each species were F = 13.57 (p<<0.001); F = 11.65 (p = 0.001) and F = 5.53 (p = 0.023) for *C. nigrifrons*, *S. nigritus* and *C. aurita*, respectively.

**Table 4:** Comparison of competing predictive models of species occurrence for *Callicebus nigrifrons*, *Callithrix aurita* and *Sapajus nigritus*, through the Akaike Information Criterion, based on occurrence information in the region of Alfenas—Brazil.

**Models**	***C. nigrifrons***		***C. aurita***		***S. nigritus***	
	**ΔAICc ^(a)^**	**wAIC ^(b)^**	**ΔAICc ^(a)^**	**wAIC ^(b)^**	**ΔAICc ^(a)^**	**wAIC ^(b)^**
Area	0	**0.476** ^1^	0	**0.378** ^1^	0	**0.454** ^1^
Area + Visibility	1.6	**0.218** ^2^	2.3	**0.120** ^4^	2.2	**0.153** ^4^
Area + Matrix	2.3	**0.154** ^3^	1.6	**0.173** ^2^	2	**0.165** ^3^
Area + Isolation	2.3	**0.151** ^4^	1.8	**0.152** ^3^	1.7	**0.196** ^2^
Visibility	14.5	<0.001	5	0.031	8.4	0.007
Isolation	15.6	<0.001	4.1	0.050	8.1	0.008
Matrix	15.9	<0.001	4.1	0.048	8.5	0.006
Matrix + Visibility	16.6	<0.001	6	0.019	10.7	0.002
Matrix + Isolation	17.8	<0.001	5.7	0.022	10.2	0.003
Null	18.8	<0.001	7.7	0.008	8.8	0.006

^(a)^ Difference between the AICc value of a given model and the lower AICc value among all models for a given species.

^(b)^ Model weight. Models were ordered following the first species in the table. For the next two species, we indicate the position of the models, from the higher to the lower weight, using superscripts.

Plausible models are highlighted.

**Table 5:** Cumulative weight of evidence (wAICc) for each individual explanatory variable of primate occurrence, obtained by the sum of the weight of models in which the variables appear.

**Metrics**	***Callicebus nigrifrons***	***Callithrix aurita***	***Sapajus nigritus***
Area	0.999	0.823	0.941
Visibility	0.218	0.17	0.162
Matrix	0.154	0.262	0.176
Isolation	0.151	0.224	0.207

**Figure 6:**
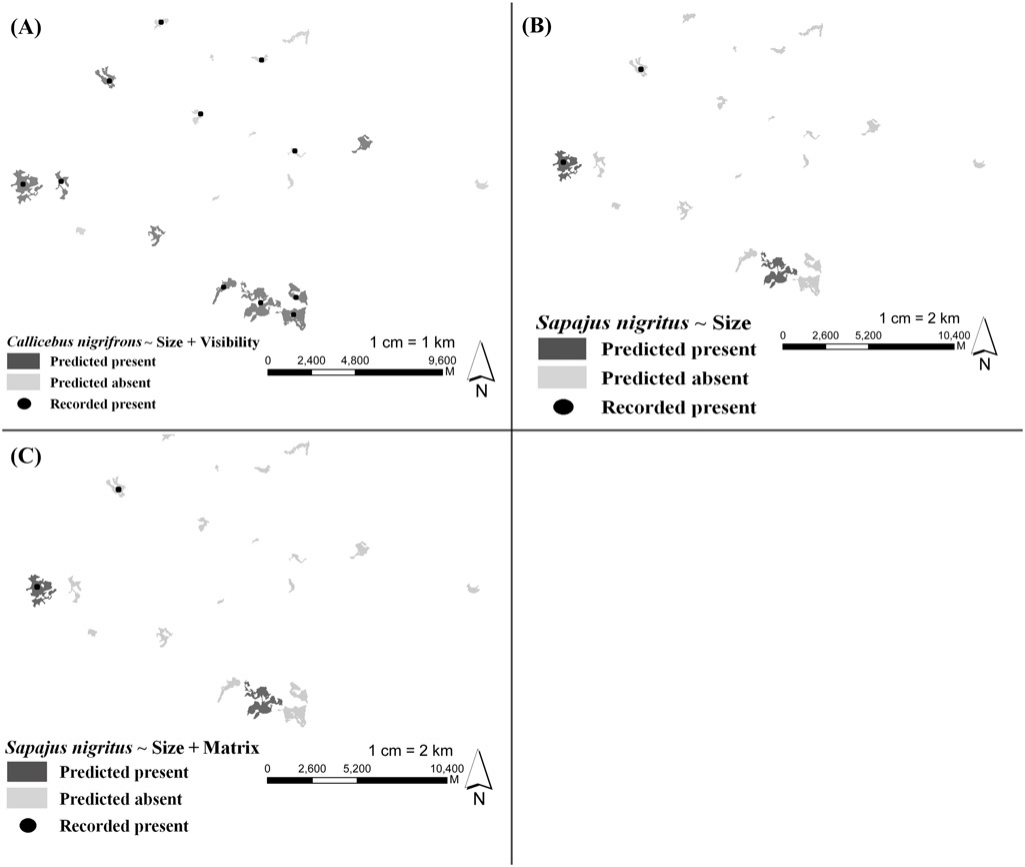
Predicted (according to plausible models that were valid according to the Kappa test) and observed primate presence/absence in the validation region, Poço Fundo—MG, Brazil. a) *Callicebus nigrifrons*—Size + visibility model; b) *Sapajus nigritus*—Size only model; c) *S. nigritus*—Size + matrix type model. Figures originally produced by LGS based on Google Earth images, for illustrative purposes only.

When validating the models with the additional 20 fragments at a neighboring locality, three of the plausible models produced significant results, with kappa>0.4 and p<0.05 (Table 6). Even the significant models ended up with several false positives and negatives (Fig. 6), something that certainly helped to decrease the kappa values.

**Table 6:** Validation of plausible models explaining the occurrence of three Neotropical primate species in the region of Alfenas, Brazil with the Kappa test, comparing prediction of species occurrence with real presence data in 20 fragments in the region of Poço Fundo, Brazil.

**Kappa statistical test**		
**Models**	**Kappa**	**(p)**
*Callicebus nigrifrons* ~ Area	0.3	0.09
*C. nigrifrons* ~ Area + Isolation	0.3	0.09
*C. nigrifrons* ~ Area + Matrix	0.3	0.09
*C. nigrifrons* ~ Area + Visibility	0.41	0.03
*Callithrix aurita* ~ Area	0.29	0.03
*C. aurita* ~ Area + Isolation	0.29	0.03
*C. aurita* ~ Area + Matrix	0.15	0.1
*C. aurita* ~ Area + Visibility	0.29	0.03
*Sapajus nigritus* ~ Area	0.44	0.02
*S. nigritus* ~ Area + Isolation	0.32	0.07
*S. nigritus* ~ Area + Matrix	0.44	0.02
*S. nigritus* ~ Area + Visibility	0.32	0.07

## Discussion

Following expectations from the theory of island biogeography [70], fragment size was crucial for explaining primate occurrence. The smaller the fragment, the smaller the probability of species occurrence, a finding also reported in other studies, both for mammals [71], and particularly for Neotropical primates [31]. However, in none of the previous studies, was the field site as degraded as ours. Around the municipality of Alfenas, there is only 4% of remaining forest cover [38], and average fragment size is 30.29ha. The size of the largest fragments in our region is, at best, equal to the size of the small fragments of similar studies [32], [72]. Populations restricted to such small remnants are extremely vulnerable to the impact of demographic, genetic and environmental stochastic factors, thus increasing the risk of local extinction [26], [73]. Hunting pressure can also have disastrous effects on very small fragments [71]. Furthermore, the smaller the fragment, the stronger are the border effects acting upon it. These factors affect, among other aspects, seed survival and germination, canopy formation and tree mortality, thus changing the structure and dynamics of the environment [74‒76]. This set of impacts related to size probably explains its emergence as the most important parameter. Since Neotropical primates depend on arboreal habitats, their survival in very small fragments is unlikely [8]. Since they provide relevant ecological services, such as predation, dispersion and frugivory, their absence may affect the entire fragment dynamic [15], [13]. However, one should note that variable fragment size, although quite important within the current set of tested variables, should by no means be considered the only explanatory variable (see, for example, [77]). The regression curves with models containing this variable alone show some data spreading, both regarding primate absence and presence. This indicates that there must be other variables (either landscape or local ones, such as fragment history or quality) that help explain the current pattern.

### Model validation

*Sapajus nigritus* was the only species for which the model with fragment size alone was validated (Table 6). Among the study species, this is the largest-bodied primate, and its groups comprise a larger number of individuals [78]. Collectively, these factors indicate the need for a larger home range for this species, thus explaining their need of larger areas. Being that fragment size is so important, why were the plausible models of other species comprised by size alone not validated? One possibility is related to the fact that the municipality of Poço Fundo, where we validated the models, has a slightly larger vegetation cover than Alfenas; thus, since fragment availability increased, the importance of fragment size decreased. Therefore, the non-validation of models including fragment size alone for the species *C. aurita* and *C. nigrifrons* may be related to the home range of these species, which are smaller than that of *S. nigritus* [43], [79].

Among the study species *S. nigritus* is also the one that travels more outside the fragments [80], [81]. This is a possible reason for the validation of the model “area + matrix permeability” for this species. This finding highlights the fact that different matrix types influence their movement, showing that matrix types that are structurally more similar to the original habitat are more permeable than structurally distinct matrix types. Since few patches in the region are large enough to provide the necessary home range even for a few groups [82], [83], the incorporation of permeable matrix types in their home range can be a crucial factor for their survival within forest fragments. Such increase in permeability can even allow sporadic access even to other fragments, thus allowing the maintenance of a metapopulation [84], [50].

Another interesting finding from this study was the validation of the model “visibility + area” for *C. nigrifrons* (Table 6). This result shows us that the visibility of a fragment surrounding a focal fragment can be positively influencing the occurrence/persistence of Neotropical primates, and possibly other mammals (or birds) as well. Such visibility can be crucial for the establishment and maintenance of primate metapopulations, given their usually high philopatric degree, with dispersal events mostly related to migration [31], [8]. This finding also shows us that the incorporation of a biologically relevant parameter in the study, reflecting the biological reality of the target species, produced interesting results.

We did not validate any of the models for *C. aurita* (Table 6), even with the species showing four plausible models (Table 4). Since the species is highly sensitive to habitat disturbance [45], we hypothesize that some other parameter, not considered in this study, may be influencing its distribution in the landscape, such as some variable related to availability of feeding resources or the degree of degradation of forest patches. The lowest weight of the variable “fragment size” among all studied species (Table 5), and the lower steepness of the regression curve (a larger data spread) confirm the notion that this variable may be less important for *C. aurita*.

Overall, we found three models that were validated, according to criteria in [69], who considers models with kappa values between 0.4 and 0.75 to be good ones. However, our kappa values are actually closer to the lower limit of such a range, once again pointing to the probable importance of other variables (from the landscape or local ones), to be ascertained in future studies.

Our study shows that the third dimension (as in the visibility parameter) and the permeability of the matrix are aspects that should be incorporated in landscape ecology studies focusing on animal conservation. The visibility methodology is simple to implement, since the necessary topographic data is easy to obtain, and the current GIS software packages are capable of working with the third dimension [35]. Moreover, we believe that the parameters of visibility and matrix permeability will show even better results for species that have a greater degree of mobility and show a lesser degree of philopatry, not being restricted to primates whatsoever. We also verified the importance of forest fragment size in the occurrence/persistence of species in highly degraded regions, a scenario in which forest cover is greatly reduced, and species persistence is even more fragile.

We hope that the successful incorporation of novel, biologically relevant metrics will inspire researchers working with similar topics to free themselves from standard metrics, and to start thinking creatively, proposing and using new, innovative metrics, which take into account the biology of their study species.

### Implications for conservation

Landscape management is crucial for the maintenance of primate populations, with the incorporation of matrix types which are more structurally similar to the original habitat, thus being more permeable, something which indirectly contributes to the dynamics of the ecological processes in the community. It is also necessary for us to consider matrix management and the visibility component in the selection of areas both for the establishment of corridors and for ecological restoration.

Future studies with these animals must aim not only at testing some of the hypotheses proposed above, but also at studying these animals in regions with a larger variation of habitat cover, on larger spatial scales (e.g. considering larger radii, of 5 or 10 km), with more rugged terrain, and with a larger matrix heterogeneity. Finally, we strongly advocate the use of matrix permeability and visibility towards neighboring fragments as important and complementary aspects of the landscape in order to understand species distribution, particularly of those species that may use their visualization range to decide when to move between habitat patches. Visibility and matrix permeability may thus be very influential aspects of the landscape for several ecological processes, including those related to gene flow and movement-modulated genetic diversity.
